# COVID‐19 associated pulmonary aspergillosis

**DOI:** 10.1111/myc.13096

**Published:** 2020-05-15

**Authors:** Philipp Koehler, Oliver A. Cornely, Bernd W. Böttiger, Fabian Dusse, Dennis A. Eichenauer, Frieder Fuchs, Michael Hallek, Norma Jung, Florian Klein, Thorsten Persigehl, Jan Rybniker, Matthias Kochanek, Boris Böll, Alexander Shimabukuro‐Vornhagen

**Affiliations:** ^1^ Department I of Internal Medicine Medical Faculty and University Hospital Cologne University of Cologne Cologne Germany; ^2^ Cologne Excellence Cluster on Cellular Stress Responses in Aging‐Associated Diseases (CECAD) University of Cologne Cologne Germany; ^3^ ZKS Köln Clinical Trials Centre Cologne Cologne Germany; ^4^ German Center for Infection Research (DZIF) Partner Site Bonn‐Cologne Medical Faculty and University Hospital Cologne University of Cologne Cologne Germany; ^5^ Department of Anesthesiology and Intensive Care Medicine Medical Faculty and University Hospital Cologne University of Cologne Cologne Germany; ^6^ Faculty of Medicine Institute for Medical Microbiology, Immunology and Hygiene University of Cologne Cologne Germany; ^7^ Institute of Virology Medical Faculty and University Hospital Cologne University of Cologne Cologne Germany; ^8^ Department of Radiology Medical Faculty and University Hospital Cologne University of Cologne Cologne Germany

**Keywords:** *Aspergillus*, ECMO, ICU, isavuconazole, SARS‐CoV‐2, voriconazole

## Abstract

**Objectives:**

Patients with acute respiratory distress syndrome (ARDS) due to viral infection are at risk for secondary complications like invasive aspergillosis. Our study evaluates coronavirus disease 19 (COVID‐19) associated invasive aspergillosis at a single centre in Cologne, Germany.

**Methods:**

A retrospective chart review of all patients with COVID‐19 associated ARDS admitted to the medical or surgical intensive care unit at the University Hospital of Cologne, Cologne, Germany.

**Results:**

COVID‐19 associated invasive pulmonary aspergillosis was found in five of 19 consecutive critically ill patients with moderate to severe ARDS.

**Conclusion:**

Clinicians caring for patients with ARDS due to COVID‐19 should consider invasive pulmonary aspergillosis and subject respiratory samples to comprehensive analysis to detect co‐infection.

## 1 INTRODUCTION

Since December 2019 coronavirus disease 2019 (COVID‐19) emerged from Wuhan City, Hubei province, China and rapidly spread around the globe becoming a pandemic threat.^1^ Risk factors for invasive pulmonary aspergillosis (IPA) are well defined in immunocompromised populations. However, patients with acute respiratory distress syndrome (ARDS) due to viral infection are prone to secondary complications like invasive aspergillosis despite lack of underlying well‐defined immunocompromising disease.^2^, ^3^, ^4^ Possible explanations for this could be an immune‐paralysis caused by viral infection‐induced ARDS and hypoxia compromising the innate host defence.^5^ In light of influenza‐pulmonary associated aspergillosis, we retrospectively analysed our patients with COVID‐19 associated ARDS in the intensive care unit (ICU) at a single centre.

## 2 PATIENTS AND METHODS

We performed a retrospective chart review of patients with COVID‐19 and ARDS admitted to the medical and surgical ICU at the University Hospital of Cologne, a 1540‐bed teaching hospital between 7 March 2020 and 22 April 2020 (Table 1).

**TABLE 1:** Patient characteristics of patients with COVID‐19 associated invasive pulmonary aspergillosis

Characteristics	Patient #1	Patient #2	Patient #3	Patient #4	Patient #5
Gender	Female	Male	Male	Male	Female
Age (y)	62	70	54	73	54
Medical history	Lap. cholecystectomy for cholecystitis Arterial hypertension Obesity with sleep apnea (BMI 31.5) Hypercholesterolemia Ex‐smoker (30 PY; 5 y previously) Moderate COPD (GOLD 2)	Vertebral disc prolapse left L4/5, flavectomy and nucleotomy Ex‐smoker (4 months previously)	Arterial hypertension Diabetes mellitus Aneurysm coiling right A. vertebralis	Arterial hypertension Bullous Emphysema Smoker Severe COPD (GOLD 3) Previous Hepatitis B	No
Underlying immuno‐compromising condition	(inhalational steroids for COPD)	No	(intravenous corticosteroid therapy 0.4 mg/kg/d, total of 13 d)	(inhalational steroids for COPD)	No
ICU Ward	MICU	MICU	SICU	SICU	MICU
ARDS					
Horowitz‐Index, admission^12^	Severe (53 mm Hg)	Severe (93 mm Hg)	Moderate (128 mm Hg)	Severe (83 mm Hg)	Moderate (167 mm Hg)
Prone positioning	Yes	Yes	Yes	Yes	Yes
vvECMO	Yes	No	No	No	No
Acute renal failure	Yes	Yes	Yes	Yes	Yes
Dialysis	No	SLEDD	SLEDD	SLEDD	No
Microbiology					
Serum GM (>0.5)	Negative	Positive (0.7)	Negative	Negative	2× positive (2.7 and 1.3)
Fungal culture	BALF: *Aspergillus fumigatus*	BALF: negative	TA: *Aspergillus fumigatus*	TA: *Aspergillus* fumigatus	TA: negative
Susceptibility testing	Azole susceptible^a^	Not applicable	Azole susceptible Itraconazole MIC 0.380 (mg/L) Voriconazole MIC 0.094 (mg/L)	Azole susceptible Itraconazole MIC 0.380 (mg/L) Voriconazole MIC 0.094 (mg/L)	Not applicable
Fungal PCR	BALF: *Aspergillus fumigatus*	BALF: *Aspergillus fumigatus*	BALF: *Aspergillus fumigatus*	TA: *Aspergillus fumigatus*	TA: negative
TA/BALF GM (>0.5)	BALF: Positive (>2.5)	BALF: Positive (>2.5)	BALF: Positive (>2.5)	Not available	Not available
Definition of IPA					
EORTC/MSG Criteria^8^	Not classifiable (no host criterion)	Not classifiable (no host criterion)	Not classifiable (no host criterion)	Not classifiable (no host criterion)	Not classifiable (no host criterion)
(modified) *AspICU* algorithm^3^, ^7^	Putative	Modified putative^3^ (GM positivity)	Putative	Putative	Modified putative^3^ (GM positivity)
Virology					
PCR	TA: positive for hMPV and SARS‐CoV‐2 (E‐gene: *C*_t_ 13.29; S‐gene: *C*_t_ 12.61)	TA: positive for hMPV and SARS‐CoV‐2(E‐gene: *C*_t_ 34.29; ORF1 a/b: *C*_t_ 31.47)	TA positive for SARS‐CoV‐2 (E‐gene: *C*_t_ 29.74; ORF1 a/b: *C*_t_ 27.86)	TA positive for SARS‐CoV‐2 (E‐gene: *C*_t_ 21.47; ORF1 a/b: *C*_t_ 20.12)	TA positive for SARS‐CoV‐2 (*C*_t_ values not available)
CT imaging studies	Combined bilateral ground‐glass opacities with crazy paving and peripheral nodular consolidations (Video S1, Figure 1A)	Ground‐glass opacities with occasional nodules (Video S2, Figure 1B)	Bilateral ground‐glass opacities, nodular infiltrates with cavities and air crescent sign (Video S3, Figure 1C)	Ground‐glass opacities with occasional nodules, known bullous emphysema (Video S4, Figure 1D)	Ground‐glass opacities, smaller areas with crazy paving pattern, central and peripheral consolidations, and smaller nodular infiltrates (Video S5, Figure 1E)
Therapy					
Antifungal treatment	Voriconazole iv (6/4 mg/kg BW twice daily)	Isavuconazole iv (200 mg thrice daily for 2 d; 200 mg once daily from 3 d)	Caspofungin (70/50 mg once daily) followed by voriconazole iv (6/4 mg/kg BW twice daily)	Voriconazole iv (6/4 mg/kg BW twice daily)	Caspofungin (70/50 mg once daily) followed by voriconazole iv (6/4 mg/kg BW twice daily)
Antiviral therapy	Supportive only	Supportive only	Hydroxychloroquine, darunavir and cobicistat at external hospital, in house changed to supportive only	Supportive only	Ribavirin, lopinavir/ritonavir at external hospital, in house changed to supportive only
Outcome	Died	Died	Alive	Died	Alive

Abbreviations: ARDS, acute respiratory distress syndrome; BALF, bronchoalveolar lavage fluid; BW, body weight; COPD, chronic obstructive pulmonary disease; *C*_t_, threshold cycle; EORTC/MSG, European Organization for Research and Treatment of Cancer/Mycoses Study Group; GOLD, global initiative for chronic obstructive lung disease; hMPV, human metapneumovirus; IPA, invasive pulmonary aspergillosis; kg, kilogram; lap, laparoscopic; LVB, lumbar vertebral body; mg, milligram; MIC, minimal inhibitory concentration; MICU, medical ICU; ORF, open reading frame; PCR, polymerase chain reaction; PY, pack‐year history; SICU, surgical ICU; SLEDD, slow low‐efficient daily dialysis; TA, tracheal aspirate; vvECMO, veno‐venous extracorporeal membrane oxygenation.

^a^ Antifungal susceptibility testing by VIPcheck™.^6^

For patient #1 SARS‐CoV‐2 RT‐PCR Kit (altona Diagnostics) and for patients #2, #3 and #4 cobas® SARS‐CoV‐2 Test (Roche) were used. For patient #5, kit manufacturer remains unknown. Respective *C*_t_ values are given in Table 1 where available. hMPV‐PCR was performed with NxTAG® Respiratory Pathogen Panel (Luminex). *Aspergillus* 28S rDNA‐Realtime PCR was performed as in‐house PCR test for screening purposes. Species identification was performed by artus® *Aspergillus* diff. RG PCR kit (Qiagen). For galactomannan testing from serum, bronchoalveolar lavage fluid (BALF) or tracheal aspirate (TA) Platelia *Aspergillus* antigen ELISA (Bio‐Rad Laboratories) was used. Antifungal susceptibility testing was performed using concentration gradient strips on RPMI agar plates. In brief, spore suspensions of the fungal isolates were adjusted to 10^6^ CFU/mL and inoculated with MIC test strips for itraconazole and voriconazole (bioMérieux). MICs were determined visually after 48 hours of incubation at 35°C. VIPcheck™ (Mediaproducts BV) was performed in patient #1 for early detection of azole resistance.^6^

COVID‐19 associated IPA was classified according to the (modified)^3^ *AspICU* algorithm^7^ with the addition that positive galactomannan (≥1) from BALF or TA, or two consecutively but separately drawn positive serum samples (≥1) were accepted as entry criterion.^8^ Severe SARS‐CoV2 infection with ARDS was accepted as host criterion (acquired immunodeficiency) (Table 1). This study was carried out in accordance with the ethical principles reflected in the Declaration of Helsinki^9^ and was approved by the ethic committee of the University Hospital of Cologne (Identifier of the University of Cologne Ethics Committee 20‐1157).

## 3 RESULTS

**FIGURE 1:**
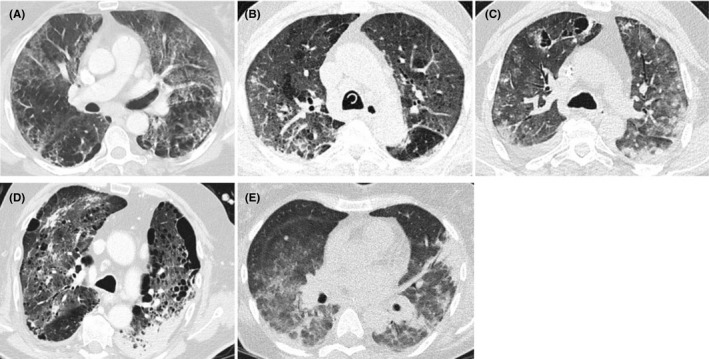
Chest CT images of patients with COVID‐19 Associated Invasive Pulmonary Aspergillosis. A, Patient #1: Combined bilateral ground‐glass opacities with crazy paving and peripheral nodular consolidations. B, Patient #2: Ground‐glass opacities with small nodular infiltrations of up to 1 cm. C, Patient #3: Bilateral ground‐glass opacities diffuse nodular infiltrates and cystic cavities and partly air crescent sign. D, Patient #4: Bullous emphysema and ground‐glass opacities, interstitial changes and consolidations with nodular infiltrates. E, Patient #5: Ground‐glass opacities, smaller areas with crazy paving pattern, central and peripheral consolidations, and smaller nodular infiltrates

A 62‐year‐old woman was admitted to our ICU. She was intubated and developed severe ARDS with a Horowitz‐Index of 53 mm Hg. At the contrast‐enhanced CT a combination of emphysema, bilateral ground‐glass opacities with crazy paving and some peripheral nodular consolidations were seen (Video S1, patient #1, Figure 1A). Despite prone positioning, the patient required rescue veno‐venous extracorporeal membrane oxygenation (ECMO). PCR for SARS‐CoV‐2 and human metapneumovirus (hMPV) was positive in BALF. The patient developed severe intrapulmonary bleeding from the right main bronchus, which was stanched by cold lavages and instillation of tranexamic acid. BALF culture grew *Aspergillus fumigatu*s, was positive for galactomannan, and intravenous voriconazole treatment was commenced.

A 70‐year‐old man was admitted to the ICU because of ARDS with a Horowitz‐Index of 93 mm Hg. PCR on BALF was tested positive for hMPV, SARS‐CoV‐2 and *Aspergillus fumigatus*. Two days before, serum galactomannan had turned positive. BALF was tested positive for galactomannan. Chest CT showed ground‐glass opacities with some small nodular infiltrations of up to 1 cm (Video S2, patient #2 Figure 1B). Due to acute renal failure requiring slow low‐efficient daily dialysis (SLEDD) and elevated liver enzymes, intravenous isavuconazole treatment was started.

A 54‐year‐old man presented with ARDS with a Horowitz‐Index of 128 mm Hg. TA revealed SARS‐CoV‐2 PCR positive and *Aspergillus fumigatus* in culture. BALF was positive for galactomannan. Chest CT showed bilateral ground‐glass opacities, diffuse nodular infiltrates and cystic cavities and partly air crescent sign (Video S3, patient #3 Figure 1C). Intravenous voriconazole treatment was initiated.

A 73‐year‐old man was transferred to the ICU due to ARDS with PCR on TA tested positive for SARS‐CoV‐2, and *Aspergillus fumigatus* that also grew in culture. Chest CT showed known bullous emphysema with ground‐glass opacities and consolidations with nodular infiltrates (Video S4, patient #4, Figure 1D). Intravenous voriconazole was begun.

A 54‐year‐old woman was transferred to the ICU due to ARDS with PCR on TA tested positive for SARS‐CoV‐2. Serum galactomannan returned positive in two consecutive serum samples. Chest CT showed bilateral ground‐glass opacities, smaller areas with crazy paving pattern, central and peripheral consolidations, and smaller nodular infiltrates (Video S5, patient #5, Figure 1E). Intravenous caspofungin was started. No autopsies were performed. Detailed patient characteristics are given in Table 1.

## 4 DISCUSSION

Patients with ARDS triggered by viral infection, in particular influenza, are prone to invasive aspergillosis even in absence of prior immunodeficiency.^2^, ^3^ We report putative IPA in five of 19 COVID‐19 patients with moderate to severe ARDS without underlying immunocompromising disease on two separate ICUs.

Spontaneous intrapulmonary bleeding and haemoptysis are typical complications of IPA. Ground‐glass opacities characterise COVID‐19 as well as IPA, thus comprehensive microbiological evaluation can prevent missing IPA.

By applying strict interpretation of the host and risk factors for invasive aspergillosis according to the European Organization for Research and Treatment of Cancer/Mycoses Study Group definitions the risk of missed diagnosis increases in the COVID‐19 population.^8^ However, a most complicated issue in ARDS patients is to differentiate *Aspergillus* colonisation from invasive disease—especially as radiological imaging is non‐specific. To address this issue, a consensus project will seek to provide standard definitions for invasive fungal disease in critically ill adult patients.^10^

Our observations suggest increased risk for critically ill COVID‐19 patients to develop co‐infection with *Aspergillus*, which is likely to increase mortality rates further. Therefore, testing for the presence of *Aspergillus* in lower respiratory secretions and galactomannan in consecutive serum samples in COVID‐19 ICU patients should be considered.^11^

Our findings need to be confirmed in clinical trials to elucidate the role of potential IPA after COVID‐19. With this report, we aim to call attention to the critical phenomenon of COVID‐19 associated IA in ARDS patients.

## CONFLICT OF INTEREST

PK has received non‐financial scientific grants from Miltenyi Biotec GmbH, Bergisch Gladbach, Germany, and the Cologne Excellence Cluster on Cellular Stress Responses in Aging‐Associated Diseases, University of Cologne, Cologne, Germany, and received lecture honoraria from Akademie für Infektionsmedizin e.V., Astellas Pharma, Gilead Sciences, GPR Academy Ruesselsheim, MSD Sharp & Dohme GmbH, and University Hospital, LMU Munich outside the submitted work. OAC is supported by the German Federal Ministry of Research and Education, is funded by the Deutsche Forschungsgemeinschaft (DFG, German Research Foundation) under Germany's Excellence Strategy—CECAD, EXC 2030—390 661 388 and has received research grants from, is an advisor to, or received lecture honoraria from Actelion, Allecra Therapeutics, Amplyx, Astellas, Basilea, Biosys UK Limited, Cidara, Da Volterra, Entasis, F2G, Gilead, Grupo Biotoscana, Janssen Pharmaceuticals, Matinas, Medicines Company, MedPace, Melinta Therapeutics, Menarini Ricerche, Roche Diagnostics, Merck/MSD, Nabriva Therapeutics, Octapharma, Paratek Pharmaceuticals, Pfizer, PSI, Rempex, Scynexis, Seres Therapeutics, Tetraphase, Vical. BWB is European Resuscitation Council (ERC) Board Director Science and Research; Chairman of the German Resuscitation Council (GRC); Member of the Advanced Life Support (ALS) Task Force of the International Liaison Committee on Resuscitation (ILCOR); Member of the executive committee of the German Interdisciplinary Association for Intensive and Emergency Medicine (DIVI); Associated Editor of the European Journal of Anaesthesiology (EJA), Co‐Editor of ‘Resuscitation’; Editor of the Journal ‘Notfall + Rettungsmedizin’. He received professional fees for lectures from the following companies: Medupdate GmbH, ‘Forum für medizinische Fortbildung (FomF)’, Baxalta Deutschland GmbH, Bayer Vital GmbH, ZOLL Medical Deutschland GmbH, C. R. Bard GmbH, GS Elektromedizinische Geräte G. Stemple GmbH, Novartis Pharma GmbH, Philips GmbH Market DACH. FD reports personal fees from Forum für medizinische Fortbildung GmbH, BioMérieux GmBH, M3 Inc, and pm‐result GmbH, outside the submitted work. FD: Associated Editor of 'BMC Anesthesiology'. DAE has no potential conflict of interest. FF reports scientific grants from the Medical Faculty of the University of Cologne (Maria Pesch grant and GEROK grant), outside the submitted work. MH reports grants from Roche, personal fees from Roche, personal fees from Roche, grants from Abbvie, personal fees from Abbvie, personal fees from Abbvie, during the conduct of the study; grants from Gilead, personal fees from Gilead, personal fees from Gilead, grants from Janssen, personal fees from Janssen, personal fees from Janssen, personal fees from Celgene, personal fees from Celgene, personal fees from Boehringer Ingelheim, personal fees from Boehringer Ingelheim, outside the submitted work. NJ reports lecture fees from Gilead, Infectopharm and MSD and travel grants from Gilead, Basilea, Correvio and Pfizer outside the submitted work. FK is supported by the German Center for Infection Research (DZIF), the German Research Foundation (CRC 1279 and CRC 1310), the Bill and Melinda Gates Foundation, and the European Research Council (ERC‐StG639961). FK received lecture and consulting honoraria from MSD, Roche and ViiV outside the submitted work. TP has no potential conflict of interest. JR has nothing to disclose. MK reports personal fees from Pfizer, Astellas Pharma, Gilead Sciences and MSD Sharp & Dohme GmbH outside the submitted work. BB is a consultant to Baxalta, Celgene, MSD, Mundipharma and received Honoraria and Research funding from Astellas, Celgene, J&J, Maquet, Miltenyi, MSD, Takeda, Roche and Sanofi. ASV reports travel grants from Gilead Sciences outside the submitted work.

## AUTHOR CONTRIBUTIONS

**Philipp Koehler:** Conceptualization (lead); Investigation (lead); Writing‐original draft (lead); Writing‐review & editing (lead). **Oliver A. Cornely:** Conceptualization (lead); Investigation (lead); Writing‐original draft (lead); Writing‐review & editing (lead). **Bernd W. Böttiger:** Investigation (supporting); Writing‐original draft (supporting); Writing‐review & editing (supporting). **Fabian Dusse:** Investigation (supporting); Writing‐original draft (supporting); Writing‐review & editing (supporting). **Dennis A. Eichenauer:** Investigation (supporting); Writing‐original draft (supporting); Writing‐review & editing (supporting). **Frieder Fuchs:** Investigation (supporting); Writing‐original draft (supporting); Writing‐review & editing (supporting). **Michael Hallek:** Investigation (supporting); Writing‐original draft (supporting); Writing‐review & editing (supporting). **Norma Jung:** Investigation (supporting); Writing‐original draft (supporting); Writing‐review & editing (supporting). **Florian Klein:** Investigation (supporting); Writing‐original draft (supporting); Writing‐review & editing (supporting). **Thorsten Persigehl:** Investigation (supporting); Writing‐original draft (supporting); Writing‐review & editing (supporting). **Jan Rybniker:** Investigation (supporting); Writing‐original draft (supporting); Writing‐review & editing (supporting). **Matthias Kochanek:** Investigation (supporting); Writing‐original draft (supporting); Writing‐review & editing (supporting). **Boris Böll:** Investigation (lead); Writing‐original draft (lead); Writing‐review & editing (lead). **Alexander Shimabukuro‐Vornhagen:** Investigation (lead); Writing‐original draft (lead); Writing‐review & editing (lead). 

## ETHICAL APPROVAL

The authors confirm that the ethical policies of the journal, as noted in the author's guideline page, have been adhered to. Our study was approved by the ethic committee of the University Hospital of Cologne (Identifier of the University of Cologne Ethics Committee 20‐1157).

## Supporting information

Video S1

Video S2

Video S3

Video S4

Video S5

Supplementary Material
