# Supplementary material for: COVID‐19 associated pulmonary aspergillosis
Source: Mycoses. 2020 May 15;63(6):528–34. doi: 10.1111/myc.13096 (PMC7267243; doi:10.1111/myc.13096)
Supplement: Supplementary file 6 — Supplementary Material [file MYC-63-528-s006.docx]

**Videos 1-5. Chest CT imaging of patients with COVID-19 Associated Invasive Pulmonary** **Aspergillosis.**

Video 1) Patient #1: Combined bilateral ground-glass opacities with crazy paving and peripheral nodular consolidations.

Video 2) Patient #2: Ground-glass opacities with small nodular infiltrations of up to 1 cm.

Video 3) Patient #3: Bilateral ground-glass opacities diffuse nodular infiltrates and cystic cavities and partly air crescent sign.

Video 4) Patient #4: Bullous emphysema and ground-glass opacities, interstitial changes and consolidations with nodular infiltrates.

Video 5) Patient #5: Ground-glass opacities, smaller areas with crazy paving pattern, central and peripheral consolidations, and smaller nodular infiltrates.
